# An Unusual Case of Right Lower Quadrant Pain: A Case Report

**DOI:** 10.5811/cpcem.2021.11.53795

**Published:** 2022-01-28

**Authors:** Sarah Jabre, Mark Supino

**Affiliations:** Jackson Memorial Hospital, Department of Emergency Medicine, Miami, Florida

**Keywords:** right lower quadrant pain, peritonitis, perforated cecal diverticulum, case report

## Abstract

**Introduction:**

The perforation of a cecal diverticulum is a rare and challenging condition for the emergency physician.

**Case Report:**

A 47-year-old man with a past surgical history of bilateral inguinal hernia repair presented to the emergency department (ED) with acute abdominal pain of three days’ duration. Pain was localized to the right lower quadrant (RLQ), with anorexia as the only associated symptom. Upon arrival to the ED, his exam demonstrated focal RLQ tenderness to palpation, rebound tenderness, and guarding. Labs did not show any elevation in inflammatory markers, liver enzymes, or lipase. Computed tomography showed no evidence of acute appendicitis, colitis, or hernia recurrence. After morphine and reassessment, the patient still had a focal peritoneal exam in the RLQ. Surgical consultation was obtained and recommended additional non-opioid analgesia as well as serial abdominal exams. After several repeat abdominal exams, there was no change in the focality of the patient’s pain. Surgery was reconsulted and opted to take the patient to the operating room for exploratory laparoscopy with “appendicitis” as the presumptive diagnosis. Pathology report revealed a perforated cecal diverticulum that was adherent to the abdominal wall. The patient did well and was discharged on his third postoperative day.

**Conclusion:**

This case further underlines that even in the era of sensitive imaging tools, the diagnostic value of a targeted physical exam with clinical re-evaluation can never be overestimated.

## INTRODUCTION

The perforation of a cecal diverticulum is a rare and potentially challenging condition for the emergency physician. It is uncommonly diagnosed in the emergency department (ED) and generally undiagnosed pre-operatively. Most patients are taken to surgery for suspected appendicitis, and only on pathology is the actual etiology discovered.^1^ We present this unusual case of abdominal pain given the absence of objective findings on workup, which could have easily led to misdiagnosis and increased patient morbidity were it not for a very convincing physical exam.

## CASE REPORT

A 47-year-old male with no past medical history and a past surgical history of bilateral inguinal hernia repairs presented to the ED with acute right lower quadrant pain of three days’ duration. Pain was constant, progressive, and associated with anorexia. There was no associated nausea, vomiting, fever, chills, dysuria, or constipation. He had never experienced any similar episodes of pain in the past. Upon arrival to the ED, his temperature was 37° Celsius, heart rate 85 beats per minute, blood pressure 114/77 millimeters of mercury, and his respiratory rate was 18 breaths per minute. On exam, his abdomen was soft and non-distended with severe, focal right-sided tenderness around McBurney’s point with associated guarding. Rovsing’s and psoas signs were negative. Coughing elicited pain in the patient, but there was no bulge suggesting recurrence of his hernia. His laboratory values and urine analysis were relatively unremarkable (Table).

**Image 1:**
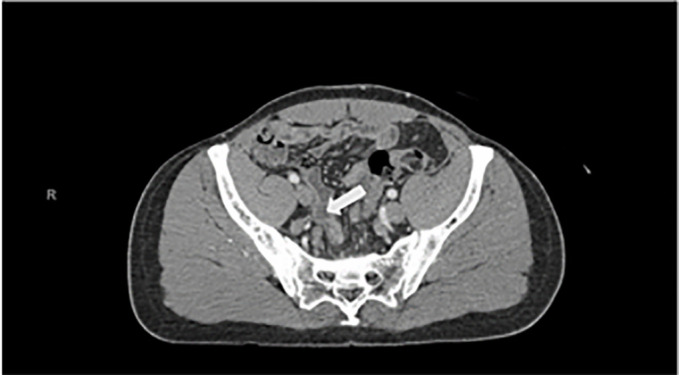
Computed tomography abdomen and pelvis with intravenous contrast with the arrow pointing to a normal appendix.

**Image 2:**
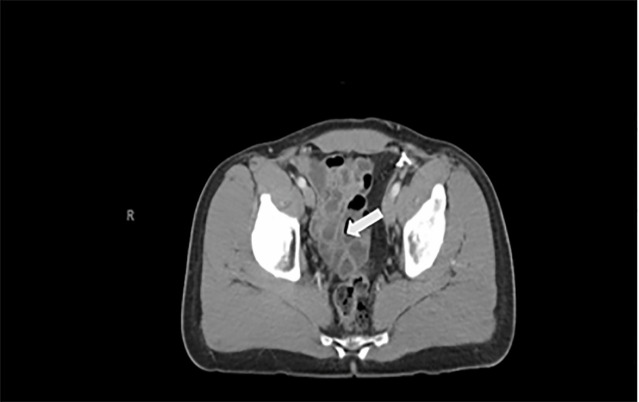
Computed tomography abdomen and pelvis with intravenous contrast with the arrow pointing to numerous fluid-filled, small bowel loops.

A computed tomography (CT) of the abdomen with intravenous contrast showed “no evidence of acute appendicitis.” The report also stated: “no evidence of recurrence of right inguinal hernia, and numerous fluid-filled small bowel loops in the right abdomen that in the appropriate setting could reflect enteritis” (Image 1 and 2).

Upon reassessment, after one dose of four milligrams of morphine, the patient still had the same exam: focal point tenderness to an area near McBurney’s point. A decision was made to consult surgery. The surgical team recommended serial abdominal exams by the emergency physician as well as administration of ketorolac as an attempt to rule out a musculoskeletal etiology of the pain. However, after serial exams six hours later, there was no change in the findings, and peritonitis remained a concern. Surgery was reconsulted and reassessed the patient, deciding to take him for exploratory laparoscopy and appendectomy, with appendicitis as the presumptive diagnosis. In the operating room, findings were consistent with cecal diverticulitis, and the surgeon removed the affected part of the cecum. The surgeon also opted to remove the appendix. Pathology reports revealed a perforated cecal diverticulum that was adherent to the abdominal wall, and a normal appendix. The patient was discharged three days postoperatively on oral antibiotics to complete a total of four days of antibiotics. He had no complications during his stay.

## DISCUSSION

Cecal diverticulosis represents only 1–2% of diverticular disease in North America with the prevalence much more common in Western countries.^2^ It is estimated that 1 in 300 cases with a preoperative diagnosis of “acute appendicitis” were in fact “cecal diverticulitis.”^3^ A review of the literature shows no definitive distinctive features between the diagnosis of the two entities. Some authors discuss a less toxic appearance or a longer duration of symptoms for people with cecal diverticulitis, but there is no clear consensus.^4^,^5^As for the management, if the diagnosis is made preoperatively through CT, conservative treatment with antibiotics is usually advised. However, if the imaging reveals evidence of complicated diverticulitis (abscess or perforation), surgery remains the modality of choice.^6^ In rare cases, right hemicolectomy or ileocolic resection is indicated.

**CPC-EM Capsule**

What do we already know about this clinical entity?

*Cecal diverticulosis represents 1–2% of diverticular disease in North America. It presents as right lower quadrant pain with no clear distinctive features*.

What makes this presentation of disease reportable?

*In this case cecal diverticulitis acted as a mimic to acute appendicitis, leading to laparoscopy that revealed a perforated cecal diverticulum*.

What is the major learning point?

*The real diagnostic tools were the history and serial physical exams, which prompted us to not rely on a falsely reassuring workup*.

How might this improve emergency medicine practice?

*Even in the age of sensitive imaging tools, we should not entirely rely on those diagnostic means to make a final diagnosis. History and physical exam remain the major tools of a good physician*.

## CONCLUSION

In the ED evaluation and workup of abdominal pain, the emergency physician should consider a perforated cecal diverticulum in an atypical presentation of right lower quadrant pain with findings that do not completely fit the clinical picture of appendicitis. It is imperative to involve a surgeon early. Finally, CT is helpful but not 100% sensitive; the clinician should keep in mind the importance of a physical exam and of clinical reevaluation to help guide final diagnosis and treatment.

## Figures and Tables

**Table:** Laboratory values obtained in the emergency department.

Test	Value	Unit	Reference Range
WBC	6.4 ×103	cells/mcL	4.0–10.5 ×103
Hb	12.3	g/dL	13.3–16.3
Platelets	282 ×103	cells/mcL	140–400 ×103
AST	31	unit/L	15–46
ALT	26	unit/L	21–72
Lactic acid	1.1	mmol/L	0.7–2.1
Creatinine	1.15	mg/dL	0.66–1.25

*WBC*, white blood cell count; *Hb*, hemoglobin; *AST*, aspartate transaminase; *ALT*, alanine transaminase; *mcL*, microliter; *g*, gram; *dL*, deciliter; *L*, liter; *mmol*, millimole; *mg*, milligram.
